# Cost-effectiveness of data driven personalised antibiotic dosing in critically ill patients with sepsis or septic shock

**DOI:** 10.1007/s10877-024-01257-9

**Published:** 2025-01-24

**Authors:** Hana M. Broulikova, Jacqueline Wallage, Luca Roggeveen, Lucas Fleuren, Tingjie Guo, Paul W.G. Elbers, Judith E. Bosmans

**Affiliations:** 1 Department of Health Sciences, Faculty of Science, Vrije Universiteit Amsterdam, Amsterdam Public Health research institute, Van der Boechorststraat 7, Amsterdam, 1081 BT the Netherlands; 2 Department of Intensive Care Medicine, Center for Critical Care Computational Intelligence, Amsterdam Cardiovascular Science, Amsterdam Institute for Infection and Immunity, Amsterdam Medical Data Science, Amsterdam Public Health, Amsterdam UMC, Vrije Universiteit, De Boelelaan 1117, Amsterdam, 1081 HV the Netherlands; 3 System Pharmacology and Pharmacy, Leiden Academic Center for Drug Research (LACDR), Leiden University, Wassenaarseweg 76, Leiden, 2333 AL the Netherlands

**Keywords:** Cost-effectiveness analysis, Model-informed precision dosing, Sepsis, Septic shock, Antibiotics, Intensive care software

## Abstract

**Purpose:**

This study provides an economic evaluation of bedside, data-driven, and model-informed precision dosing of antibiotics in comparison with usual care among critically ill patients with sepsis or septic shock.

**Methods:**

This economic evaluation was conducted alongside an AutoKinetics randomized controlled trial. Effect measures included quality-adjusted life years (QALYs), mortality and pharmacokinetic target attainment. Costs were measured from a societal perspective. Missing data was multiply imputed, and bootstrapping was used to estimate statistical uncertainty. Differences in effects and costs were estimated using bivariate regression and used to calculate incremental cost-effectiveness ratios.

**Results:**

Patients in the intervention group had higher costs (€42,684 vs. 39,475), lower mortality (42% vs. 49%), more QALYs (0.184 vs. 0.153), and higher pharmacokinetic target attainment (69% vs. 48%). Only the difference for target attainment was found statistically significant. An additional €18,129, €55,576, and €123,493 needs to be invested to attain the targeted plasma levels for one more patient, to save one life and gain one QALY, respectively. The probability of cost-effectiveness for all effect outcomes is below 60% for most acceptable willingness-to-pay thresholds.

**Conclusions:**

Data-driven personalised antibiotic dosing in critically ill patients as implemented in the AutoKinetics trial cannot be recommended for implementation as a cost-effective intervention.

**Trial registration:**

The trial was prospectively registered at Netherlands Trial Register (NTR), NL6501/NTR6689 on 25 August 2017 and at the European Clinical Trials Database (EudraCT), 2017-002478-37 on 6 November 2017.

**Supplementary Information:**

The online version contains supplementary material available at 10.1007/s10877-024-01257-9.

## Introduction

Sepsis and septic shock affect about 50 million patients worldwide every year [1, 2]. In critically ill patients with sepsis, mortality rates remain at 15–30% or even higher despite treatment in intensive care units (ICUs) [3]. Among survivors, the personal and economic burden of sepsis is also enormous as it entails impaired quality of life, increased healthcare consumption and increase in long-term mortality [4]. 

The mean total costs of a hospital stay for patients with sepsis amounts to €26,402 in the United States and €30,475 in Europe (both 2020 prices) [5]. Costs of the initial hospitalization for acute sepsis account for about 30% of all costs. The rest is distributed between follow-up healthcare costs and productivity losses [6–8]. Due to the higher daily costs and longer duration of treatment, the costs of intensive care admissions are about 2.5 times higher in patients with sepsis than among patients with other conditions that require intensive care [9–11]. 

To treat sepsis, fast and adequate administration of antibiotics is crucial [12]. Their early and appropriate use has been associated with improved clinical outcomes [13, 14]. Overdosing of antibiotics may contribute to toxicity induced morbidity while underdosing may lead to suboptimal treatment outcomes and antimicrobial resistance [15–19]. However, achieving and maintaining adequate antibiotic levels is challenging, especially in the critically ill. Despite markedly altered and rapidly changing pharmacokinetic (PK) profiles of patients, antibiotics typically continue to be dosed following standard regimens. This suboptimal practice may be related to insufficient knowledge of PK among intensive care professionals [16]. 

We developed software to assist ICU professionals with antimicrobial dosing called AutoKinetics. AutoKinetics provides model-informed predictions and graphically displays patient-specific antibiotic plasma concentrations together with the corresponding dosing advice in real-time [20, 21]. As a result, it reduces the need for manual data entry and guidance by pharmacists and facilitates precision dosing of administered antibiotics. A two-center randomized controlled trial was performed to assess feasibility, safety, efficacy, and cost-effectiveness of clinical implementation of AutoKinetics. The feasibility, safety and efficacy results were published elsewhere [22]. This study provides economic evaluation of bedside, data-driven, and model-informed precision dosing by AutoKinetics in comparison with usual care among critically ill patients with sepsis or septic shock.

## Methods

### Study design

We performed a cost-effectiveness analysis alongside the AutoKinetics randomized controlled trial. This was an investigator-initiated, two-center, non-blinded superiority trial with two parallel arms conducted at Amsterdam UMC, location VUmc and OLVG, location East in Amsterdam, the Netherlands. Both are tertiary referral centers with medical and surgical patients. Ethical approval was obtained in both centers (VUmc 2017.474, OLVG 18.011) and the trial was monitored by an independent clinical research bureau [21]. 

All adult intensive care patients were eligible for inclusion if they received antibiotics for a suspected or confirmed infection and had a suspected or measured serum lactate greater than two mmol/L or a requirement for vasopressor support in any dose. Patients were eligible for inclusion both at the start of and during their antibiotic course as soon as the lactate or vasopressor criterium was reached. There were no exclusion criteria. To avoid any delays in treatment, deferred consent was obtained from patients or their representatives within 48 hours of randomization.

### Interventions

In the AutoKinetics group, the individualized dose and dosing frequency recommendations for a predefined PK dosing target were instantly available at the bedside for up to four (one primary and up to three secondary) antibiotics used in the two participating centers: the β-lactam antibiotics ceftriaxone and meropenem, the fluoroquinolone ciprofloxacin, and the glycopeptide vancomycin [22]. Physicians had the option to accept or decline the system’s dosing recommendations and compliance was monitored. The AutoKinetics software combines population PK models with relevant available electronic health records data. The software is integrated with two frequently used electronic medical record systems (MetaVision, iMDsoft, Tel Aviv, Israel and Epic, Epic Systems, Verona, WA, United States). For each antibiotic, best performing PK models were selected, validated, calibrated, and implemented in AutoKinetics, as published previously [20]. 

In the control group, patients received antibiotics according to standard dosing regimens in each hospital, in line with international standards. Details on dosing, plasma sampling and training of the ICU staff in this trial were published previously [22]. 

### Outcomes

The outcome for the cost-utility analysis was Quality-Adjusted Life-Years (QALYs). QALYs were measured with the EQ-5D-5L questionnaire [23]. The Dutch EQ-5D-5L tariff was used to convert EQ-5D-5L health states to utility scores [24]. Because patients were unable to fill out the EQ-5D-5L at baseline, the utility value at baseline was approximated based on expert opinion by assuming response level 5 in the domains of mobility, selfcare and daily activities, and 3 in the domains of pain and anxiety. The resulting utility value 0.153 was assumed for all included patients. The EQ-5D-5L questionnaire was administered at two points during follow-up: at discharge from the ICU, and 6 months after ICU discharge. Linear interpolation between the different time points was used to estimate QALYs acquired during the whole period from ICU admission to 6 months after ICU discharge.

For the cost-effectiveness analysis, the respective outcomes were mortality at 6 months after the ICU discharge, and PK target attainment (TA) during the first 24 hours following randomization for the primary antibiotic. The TA endpoint was prespecified at 75% of the dosing target, which can be considered a conservative clinical end point [25]. 

Costs were assessed from a societal perspective and included: (a) admission days in ICU and in a general ward following ICU discharge which was obtained from hospital records, (b) healthcare utilization following hospital discharge measured with the iMTA Medical Consumption Questionnaire (iMCQ) [26], and (c) productivity losses measured with the iMTA Productivity Cost Questionnaire (iPCQ) [27]. The iMCQ questionnaire asks about the utilization of a wide range of the medical, paramedical, and home help services as well as medication in the past 3 months. The iPCQ asks about presenteeism and absenteeism related to paid work, and absenteeism from voluntary work in the past 4 weeks. Both questionnaires were administered at the end of the follow-up period (i.e., 6 months after ICU discharge).

Healthcare utilization as well as hours of presenteeism and absenteeism from paid work and absenteeism from voluntary work were multiplied by the unit costs according to the Dutch costing manual [28]. Costs based on the questionnaires were linearly extrapolated to cover the 6-month period, excluding days spent at a general hospital ward after ICU discharge. Healthcare follow-up costs as well as productivity losses were considered only for patients who survived until discharge from the hospital (i.e., ICU and subsequent ward discharge). Proportional healthcare follow-up costs and productivity losses were considered for those who died before the end of the follow-up period. All reported costs are in 2020 prices.

### Statistical analysis

The cost-utility and cost-effectiveness analyses were conducted from the societal perspective. The follow-up of this study was 6 months after discharge from the ICU, with baseline being defined as the timepoint at which the participant was admitted to the ICU. In accordance with the effectiveness analysis [22], the analysis was adjusted for age, gender, and hospital where the patient was treated. In addition, a crude sensitivity analysis (SA1) and sensitivity analysis adopting healthcare perspective (SA2) were performed for each outcome. The healthcare perspective included costs incurred in the hospital (ICU and follow-up ward) and the healthcare costs during the 6 months after discharge. All analyses were performed using R programming software version 4.0.2 and RStudio version 2022.07.1.

Multiple imputation with chained equations in combination with predictive mean matching was used to impute missing values [29]. The imputation was stratified by the survival status at ICU discharge and at 6 months, leading to three different patient groups: those who survived the follow-up, died after ICU discharge, died at the ICU. Although stratification for different treatment groups is preferred in the imputation [30], treatment group was included as a predictor in the imputation model, because further stratification beyond survival status would lead to low numbers of people in each stratum. For patients who died during ICU admission, quality of life for the period after death, follow-up healthcare costs, and lost productivity costs were assumed to be zero. For patients who died after being discharged from the ICU, no follow-up cost and utility data was collected. For these patients, we assumed zero utility after death and calculated costs by multiplying the average daily costs of survivors with the number of days survived after ICU discharge.

Disaggregated cost differences between the intervention (AutoKinetics) and control (usual care) group were estimated using crude linear regression models. Differences in effects and total costs were estimated using bivariate regression analysis, which accounts for the potential correlation between costs and effects [31]. Incremental Cost-Effectiveness Ratios (ICER) were calculated by dividing the difference in costs between the treatment and control group by the difference in QALYs, 6-month mortality and TA. Bootstrapping with 5,000 repetitions was used to estimate 95% confidence intervals around the cost differences and the uncertainty surrounding the ICERs which is presented using cost-effectiveness planes [32]. Cost-effectiveness acceptability curves were estimated to show the probability that AutoKinetics is cost-effective compared to usual care for a range of willingness to pay values [33]. Since there is no consensus on the willingness to pay threshold for the TA outcome, we set the threshold to the costs of one, five and ten days at the ICU.

## Results

### Participants

Between 2 February 2018 and 20 March 2020, a total of 349 patients were enrolled in the trial in both centers. Of these patients 50 patients were excluded due to failure to obtain informed consent within pre-specified time window, and 47 patients refused to participate. Informed consent was obtained for 252 patients: 132 (68% men, median age 67 years, interquartile range 58–75) were randomized to the AutoKinetics group and 120 (68% men, mean age 66 years) to the control group. In the AutoKinetics group, less than 2% of dosing recommendations was rejected by the clinicians. The trial was stopped early due to the COVID-19 pandemic with about 75% of intended inclusions. A more detailed description of the enrolment process, study population and its baseline characteristics can be found in the clinical effectiveness paper [22]. 

### Cost-effectiveness analysis

Table 1 shows the mean costs and effects incurred by the two treatment groups and its differences. Supplementary tables S1 and S2 show days spent at ICU and a general ward, and costs stratified according to the survival status. The societal costs were higher in the AutoKinetics group (€42,684 vs. 39,475). This is caused by the higher healthcare costs that outweigh the lower productivity losses in the AutoKinetics group. At the end of follow-up, 56 (42%) and 59 (49%) of deaths occurred in the AutoKinetics and control group, respectively. Patients in the AutoKinetics group experienced more QALYs (0.184 vs. 0.153) and had higher target attainment (69% vs. 48%). Except for the TA outcome, neither differences in costs nor effects were statistically significant.

**Table 1:** Costs and effects in the two treatment groups

	AutoKinetics (*N* = 132) Mean (SE)	Control (*N* = 120) Mean (SE)	Difference (CI)
Cost category			
Total healthcare costs	40,712 (1,390)	37,141 (1,210)	3,571 (-7,101 to 15,981)
*Hospital costs*	34,164 (1,272)	31,790 (1,091)	2,373(-7,161 to 13,882)
*Follow-up costs*	6,548 (412)	5,351 (383)	1,198 (-2,330 to 4,618)
Lost productivity costs	1,972 (114)	2,334 (159)	-362 (-1,666 to 757)
Total societal costs	42,684 (1,429)	39,475 (1,258)	3,209 (-7,835 to 16,009)
Effect outcomes			
Deaths	0.424 (0.014)	0.492 (0.014)	-0.067 (-0.191 to 0.056)
QALY	0.184 (0.005)	0.153 (0.005)	0.031 (-0.015 to 0.077)
TA	0.689 (0.012)	0.475 (0.014)	0.214 (0.095 to 0.334)

Legend: SE - standard error, CI – 95% confidence interval, QALY – quality-adjusted life years, TA - pharmacokinetic target attainment

Table 2 shows the results of the cost-utility (outcome QALY) and cost-effectiveness (outcome death prevented, and TA) analysis. In all analyses, the AutoKinetics intervention was on average more effective and more costly than control, although there was considerable uncertainty. To gain one QALY, an investment of €123,493 is required. The investment of €55,576 and €18,129 is required to prevent one death and attain the PK target for one more patient, respectively. The required amounts slightly drop in the crude analysis and increase when the healthcare perspective is adopted.

**Table 2:** Results of the cost-utility and cost-effectiveness analysis

Analysis	Perspective	Outcome	D Effect	D Cost	ICER	NW (%)	NE (%)	SW (%)	SE (%)
Adjusted	Societal	QALY	0.031 (-0.015 to 0.077)	3,855 (-7,209 to 16,601)	123,493	5	69	3	23
SA1 Crude	Societal	QALY	0.031 (-0.015 to 0.077)	3,209 (-7,835 to 16,009)	104,096	5	65	4	26
SA2 Adjusted	Healthcare	QALY	0.031 (-0.015 to 0.077)	4,202 (-6,460 to 16,539)	134,617	6	71	3	20
Adjusted	Societal	Mortality*	0.069 (-0.053 to 0.192)	3,855 (-7,209 to 16,601)	55,576	7	67	6	20
SA1 Crude	Societal	Mortality	0.067 (-0.056 to 0.191)	3,209 (-7,835 to 16,009)	47,588	7	63	7	23
SA2 Adjusted	Healthcare	Mortality	0.069 (-0.053 to 0.192)	4,202 (-6,460 to 16,539)	60,582	8	69	5	18
Adjusted	Societal	TA	0.213 (0.096 to 0.330)	3,855 (-7,209 to 16,601)	18,129	0	74	0	26
SA1 Crude	Societal	TA	0.214 (0.095 to 0.334)	3,209 (-7,835 to 16,009)	14,966	0	70	0	30
SA2 Adjusted	Healthcare	TA	0.213 (0.096 to 0.330)	4,202 (-6,460 to 16,539)	19,762	0	77	0	23

Legend: * Expressed as prevented deaths at 6 months (i.e., multiplied by -1), SA – sensitivity analysis, QALY – quality-adjusted life years, TA - pharmacokinetic target attainment, ICER – Incremental Cost-Effectiveness Ratios, NW – northwest, NE – northeast, SW – southwest, SE – southeast

**Fig. 1:**
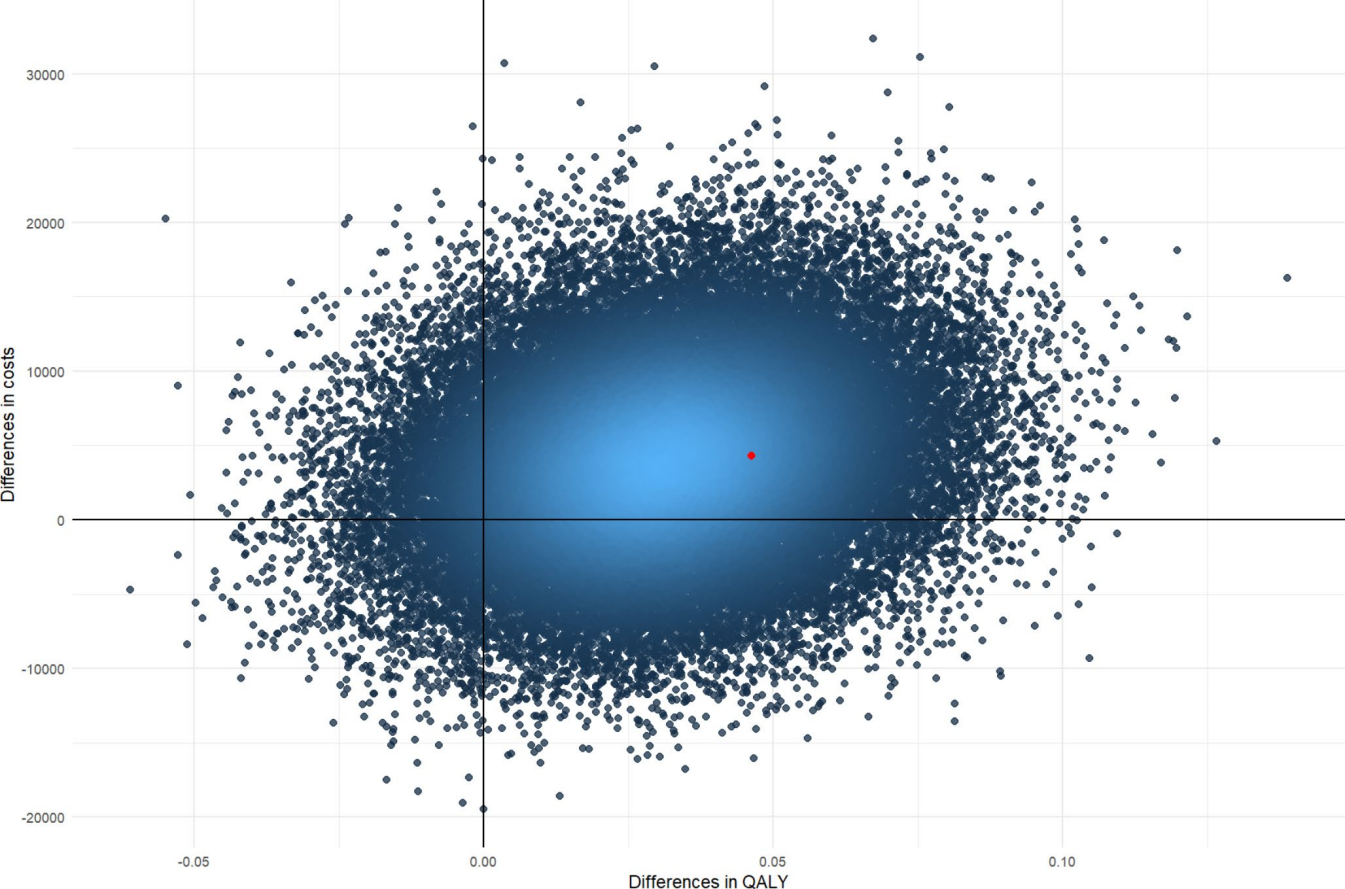
Cost-effectiveness plane for the adjusted analysis with quality-adjusted life years (QALY) as an outcome assessed from the societal perspective

**Fig. 2:**
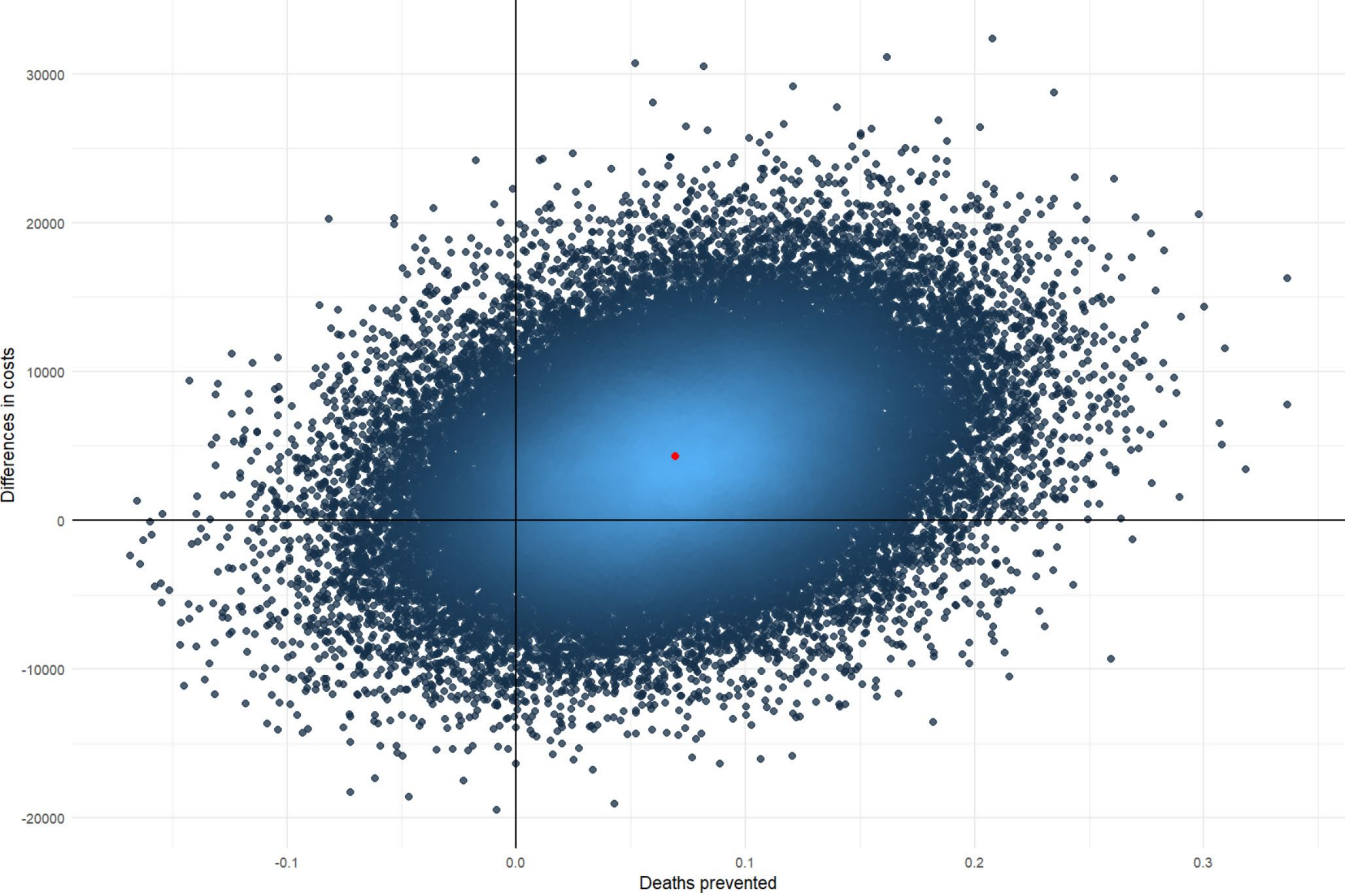
Cost-effectiveness plane for the adjusted analysis with prevented death as an outcome assessed from the societal perspective

**Fig. 3:**
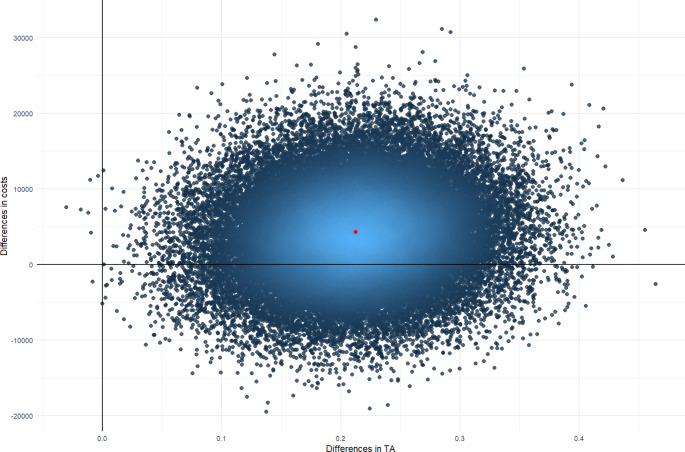
Cost-effectiveness plane for the adjusted analysis with pharmacokinetic target attainment (TA) as an outcome assessed from the societal perspective

Uncertainty around ICERs is presented using cost-effectiveness planes. Figures 1, 2 and 3 depict cost-effectiveness planes for the adjusted analysis. For all outcomes, the largest proportion (67–74%) of the bootstrapped cost-effect pairs falls in the north-east quadrant indicating that AutoKinetics is more costly, but also brings (slightly) more health effects. In this quadrant, a decision needs to be made whether society is willing to pay such amounts of money for one additional QALY, one prevented death or a TA reached in one extra patient.

**Fig. 4:**
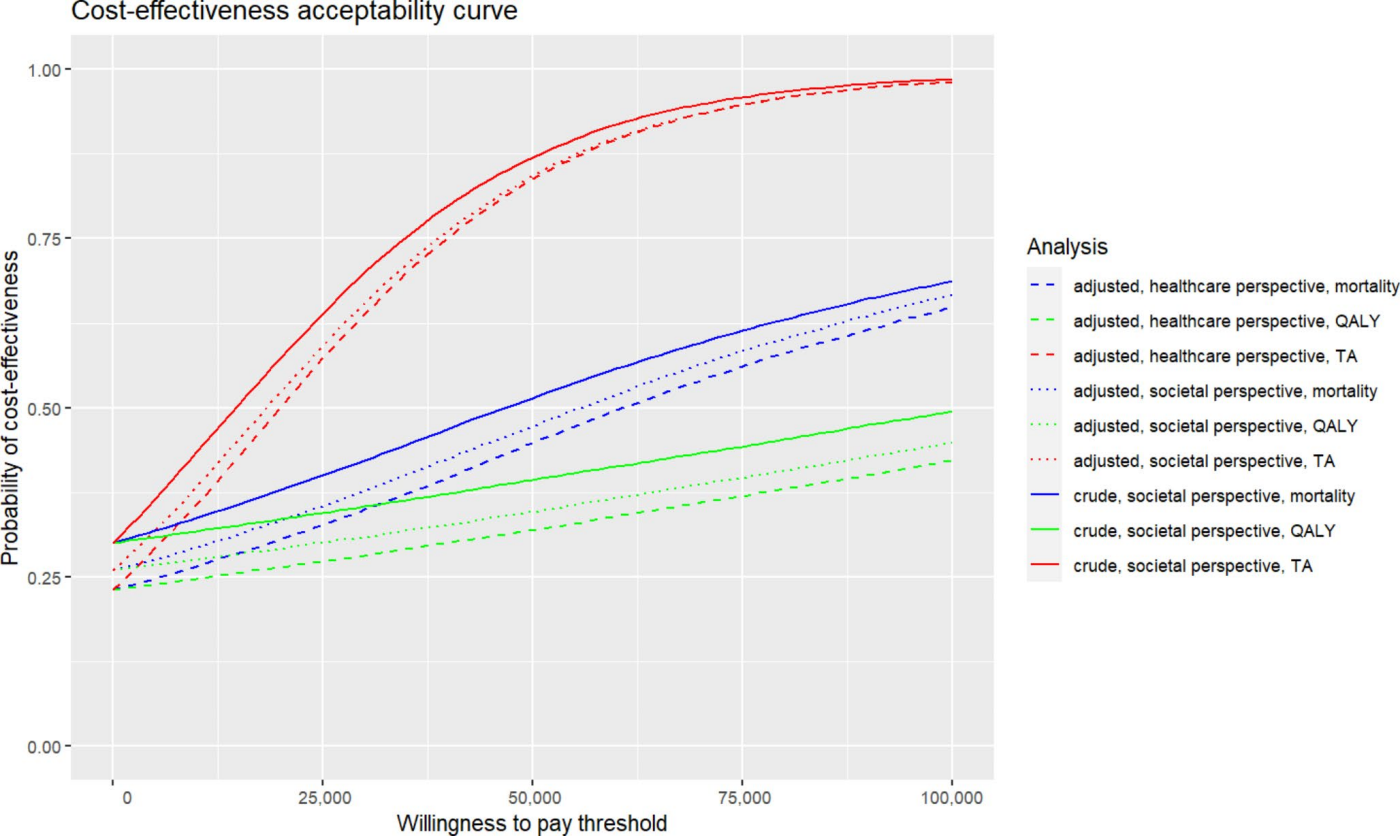
Cost-effectiveness acceptability curves for all evaluated analyses. Legend: QALY – quality-adjusted life years, TA - pharmacokinetic target attainment

Figure 4 shows probability of cost-effectiveness for different values of the willingness to pay thresholds using cost-effectiveness acceptability curves for all nine analyses. When QALYs and death at 6 months are considered as outcomes, the adjusted analysis from the societal perspective estimates a probability of the intervention being cost-effective of 35/47% for the threshold €50,000, and 41/60% for the threshold €80,000. For the TA outcome, the respective probability of being cost-effective is 28%, 38% and 52% for the threshold €2,000, 10,000 and 20,000. Since there is no consensus on the willingness to pay threshold for the TA outcome, we selected a cost of spending one, five and ten days at the ICU.

## Discussion

Our results show that although differences were not statistically significant, on average patients in the AutoKinetics group have higher costs (€42,684 vs. €39,475) and better outcomes than patients in the control group. Only the difference in TA between groups was statistically significant. The difference in total costs is driven by longer ICU admissions (11.8 vs. 10.1 days or €25,695 vs. €22,050) and higher medical follow-up costs (€6,540 vs. €5,351) in the AutoKinetics in comparison to the control group. However, people in the AutoKinetics group spent a shorter time at the general ward (12,2 vs.14.1 days or €8,514 vs. €9,741) making the total time spent in the hospital comparable between the groups (24.1 vs. 24.2 days). Thus, the higher hospital costs in the AutoKinetics group can be explained by the fact that a day spent at ICU is more expensive than a day spent at a general ward (€2,176 and €693 per admission day, respectively). The difference in total societal costs is partly mitigated by lower productivity losses among people in the AutoKinetics group in comparison to the control group (€2,334 vs. €1,972). Since productivity losses are not considered in the sensitivity analysis adopting the healthcare perspective, the larger difference in (healthcare) costs results in a slight increase in ICERs. Given the low probability of cost-effectiveness for the most acceptable willingness to pay thresholds for all outcomes, the AutoKinetics cannot be currently recommended for implementation as a cost-effective intervention.

Compared with a previously published estimate of hospital stay costs among patients with sepsis (€30,475) [5], the mean costs of a hospital stay in our study are slightly higher in the AutoKinetics group (€34,164), but similar in the control group (€31,790) The total length of stay in the hospital for patients in both arms of our trial (24 days) is in line with results previously reported from France and Germany [10, 11]. In contrast, patients with severe sepsis in the US were previously found to stay in a hospital about a week shorter [9]. The length of ICU stay in the published studies from France and the US was comparable with the control group of our trial (10 days) [9, 10]. Patients in the AutoKinetics group spent about two days more at the ICU. We further found higher costs in survivors than non-survivors (total/hospital/ICU costs €54,852/40,558/26,921 vs. €24,935/24,170/20,281, average for both treatment groups). This result is in line with a British study (survivors €25,189 vs. non-survivors €16,753, only hospital costs considered) [34]. In contrast, two French studies showed the opposite for both the total hospital costs (€36,952 vs. €39,569) [35], as well as ICU costs only (€23,775 vs. €29,132) [10]. 

This study is the first to evaluate cost-effectiveness of bedside, model-informed precision antibiotics dosing in critically ill patients with sepsis or septic shock. The study is based on a pragmatic trial, which included a representative population of critically ill patients with sepsis or septic shock. The societal perspective adopted by this study allows for the identification of potential shifts between budgets and is another important strength. However, there are a few important limitations of this study to be considered as well. First, the trial lacked power to detect relevant differences in outcomes. Especially for costs, larger sample sizes are needed given the skewed distribution of costs [36]. This is partly a design choice, because the trial was primarily designed as a safety and feasibility study given the novelty of bedside data driven antibiotic dosing. More importantly, patient inclusion was stopped prematurely after inclusion of approximately 75% of the intended inclusions due to the Covid-19 pandemic. Second, the EQ-5D-5L questionnaires were administered at ICU discharge and at the end of follow-up. The resulting utility scores were interpolated between these timepoints. However, people are still very sick at discharge from ICU resulting in low utility scores. Before they are discharged from the hospital, their utility scores probably increase considerably. Thus, the estimated QALYs are probably an underestimation of the actual QALYs. This disproportionately impacts the AutoKinetics group, since survival is higher in this group than in the control group. Third, differences in target attainment were only reached for one of the four antibiotics studied. Potential explanations for the differences among classes of antibiotics include the relatively poor performance of some pharmacokinetic models used. For clinical practice, better PK models need to be developed [22]. Additionally, the flexible timing of the intervention relative to the initiation of antibiotic therapy and the possibility of multiple concurrent antibiotic courses may have blurred the clinical benefit and effectiveness of each antibiotic course [22]. These three limitations together likely explain the large confidence intervals surrounding the point estimates of the outcomes. Fourth, we considered productivity costs of those who died as equal to 0. This assumption leads to the situation where people who survived could incur lost productivity costs, whereas those who died do not. This assumption specifically affects the AutoKinetics group, because survival in this group was higher than in the control group. On the other hand, most people in the sample were likely out of the work force due to the high age (60% aged over 65 years). Consequently, it is unlikely that the results are seriously biased by this assumption. Fifth, at this stage of development, the future costs of the AutoKinetics software are unknown. Due to uncertainty regarding both the software’s price and the number of patients at the ICU who could benefit from acquiring the software, we have decided against specifying any unit cost per patient. Finally, the rate of missing data was high, amounting to 44% for QALYs and 50% for costs. We used multiple imputation to impute missing values which is the recommended method to use. Instead of stratifying the imputation model by treatment group as recommended, we stratified by survival status only, because further stratification would result in too small numbers per stratum.

Future research should address the limitations mentioned above to validate our findings and address the uncertainty around results. Specifically, the sample size needs to be large enough to detect significant differences in effects and costs, health-related quality of life should be measured at all important timepoints such as being discharged from the hospital, and the missingness in the data should be reduced. Of note, the intervention group is disproportionately affected by the study limitations. Addressing them could, consequently, lead to more favorable cost-effectiveness results.

## Conclusion

Given the low probabilities of cost-effectiveness for QALYs and 6-month mortality outcomes, the AutoKinetics software with the currently implemented pharmacokinetic models cannot be at present recommended for implementation. Although there was a statistically significant effect of the intervention on the pharmacokinetic target attainment, consensus on the willingness to pay threshold for this outcome does not exist, which hinders interpretation of the respective cost-effectiveness acceptability curve.

## Electronic Supplementary Material

Below is the link to the electronic supplementary material.

Supplementary Material 1

## Data Availability

Data can be made available upon reasonable request considering applicable regulations and legislation.
