# Supplementary material for: Cost-effectiveness of data driven personalised antibiotic dosing in critically ill patients with sepsis or septic shock
Source: J Clin Monit Comput. 2025 Jan 24;39(5):937–46. doi: 10.1007/s10877-024-01257-9 (PMC12474602; doi:10.1007/s10877-024-01257-9)
Supplement: Supplementary file 1 — Supplementary Material 1 [file 10877_2024_1257_MOESM1_ESM.docx]

**Appendix**

*Table S1 Mean costs according to the treatment group and survival status*

| Cost category | Control | | | AutoKintetics | | |
| --- | --- | --- | --- | --- | --- | --- |
|  | All | Survivors | Non-survivors | All | Survivors | Non-survivors |
| Hospital | 31,790 | 40,950 | 22,320 | 34,164 | 40,165 | 26,019 |
| *Intensive care unit* | 22,050 | 25,755 | 18,219 | 25,650 | 28,087 | 22,342 |
| *Ward* | 9,741 | 15,195 | 4,101 | 8,514 | 12,078 | 3,677 |
| Medical follow-up | 5,351 | 9,942 | 604 | 6,548 | 10,997 | 510 |
| Productivity | 2,334 | 4,337 | 263 | 1,972 | 3,311 | 154 |
| Total societal | 39,475 | 55,229 | 23,187 | 42,684 | 54,474 | 26,683 |

*Table S2 Mean days spent in the hospital according to the treatment group and survival status*

| Days category | Control | | | AutoKintetics | | |
| --- | --- | --- | --- | --- | --- | --- |
|  | All | Survivors | Non-survivors | All | Survivors | Non-survivors |
| Hospital | 24.2 | 33.8 | 14.3 | 24.1 | 30.3 | 15.6 |
| *Intensive care unit* | 10.1 | 11.8 | 8.4 | 11.8 | 12.9 | 10.3 |
| *Ward* | 14.1 | 21.9 | 5.9 | 12.3 | 17.4 | 5.3 |
